# Influenza Specifics

**Published:** 1892-01-16

**Authors:** 


### The Hospital

A Weekly Journal of

Science, Medicine, Nursing, and Philanthropy.

*For Hospitals, Asylums, Infirmaries, Dispensaries, Medical Practitioners, Students, Nurses, and the Charitable Public.*

Vol. XI.—No. 277.] [Jan. 16, 1892.

### Influenza Specifics.

The public shows great faith in medicine when it looks to the members of that profession for specifics for influenza. It is not too much to say that such a thing as an absolute specific for any single disease is altogether unknown. The value of any particnlar medical man may be very well estimated by the degree of faith he professes in specifics. Dr. Pfeiffer informs us that he has found the influenza microbe, and every daily paper has been full of the "cinnamon" cure. No microbe of any kind, we are told, can stand against cinnamon. How delightful this is! All that the patient who is attacked with influenza has to do is to put a little cinnamon on the tails of all the microbes, and the disease will vanish like a flash of lightning. The doctor who has the task of extinguishing these airy hopes by a little plain truth feels that he is a grim kind of ogre whom everybody must detest. Indeed, the sober practitioner, in these days of "cure-made-easy," is conscious that he is little better than a mere skid on the wheels of more dashing men. He thinks he knows that the "dasher" is more or less of a mountebank, and that the public will find out his mountebank's tricks bye and bye. In the meantime, however, he is painfully aware that the mountebank has the popular ear, and that mere sober sense is looked upon as bronze, whilst the pretender's sham notes are everywhere promptly exchanged for the golden coin of the realm.

Is it the case, then, that medical men can do nothing at all for influenza patients? The question is a little absurd. But it is the kind of question which is constantly presenting itself to the doctor's mind when he hears it so persistently suggested that medicine is of no use if it cannot in so long a time as two or three years discover a specific for so comparatively mild an epidemic disease as influenza. What are the doctors worth, asks the cocksure young man of the provincial weekly newspaper, if they cannot tell us what to do to avoid influenza, or if they cannot all be of one mind about the treatment when once the disease has appeared in a patient? To which we answer that when the millennium arrives doctors will no doubt be able to put their fingers on a disease on the very first day of its appearance, and to prescribe a specific which will cure it in a moment, just as the conjuror produces a chicken out of a hat in which he had placed the very moment before a new-laid egg. Only one would suggest that if specifics are to be so general and so prompt in action as appears to be universally expected, nature has made a mistake in producing any diseases at all. The business of disease and its treatment, when specifics shall have reached the stage of perfection which we have prefigured, will be reduced to such a simple art as that of setting up nine-pins for the mere pleasure of knocking them down again.

It is exceedingly curious to observe how primitive human instincts still assert themselves, even among people who are bred in old civilizations. "The heirs of all the ages, in the foremost files of time," have much the same idea about medical science and practice as Naaman the Syrian had when he was so disgusted with the prophet because the latter did not come forth from his abode with the air of a magician, strike his hand over the great man's leprosy, make incantations to his God, and cure the disease at a single miraculous stroke. The Central African savages are in a similar state of mind to-day to that of the Syrian commander-in-chief of three thousand years ago. But we think we have a right to expect better things of the scientifically-educated people of our own times. If it were only the very poor, or even those trained at Board schools, who believed in wonders, specifics, and quack pretenders, one would not be so much surprised. But when one finds journalists, Members of Parliament, and society men and women just as ignorant of the problems that are involved in the nature and management of disease, and just as ready to pin the most child-like faith to persons who are manifestly ignorant pretenders, as are the untaught savage and the unscientific denizen of the slums, one may be excused for feeling that this also is one of those human mysteries which imperatively demand solution.

Has medicine, then, nothing of a practical kind to say to those who are suffering from influenza, and to those who are dreading its attack? It has much to say to both; but it expects to find in both a reasonable amount of intelligence. For the comfort of men and women who are already in the jaws of the devourer, it is important to state that all intelligent medical men now understand the nature and course of influenza very well, and are generally agreed as to the leading principles of its treatment. They know, for example, that it is absolutely necessary for every patient to be put to bed, and to be actively treated and intelligently nursed. They know also this very important principle, that depressants are to be avoided, and that stimulants and tonics are to be administered. They recognise thoroughly that one of the most important details of treatment is that of the feeding of their patients. Whether a person suffering from influenza have an appetite or not, it is absolutely imperative that he be frequently fed with nutritious food. All this the doctor knows well, and he knows, moreover, a fact which is of the first importance, that in ordinary cases of influenza a nutritious diet is well tolerated and produces no injurious consequences.

We are in this condition of things with regard to influenza, that though we have no specific for it which "acts like a charm," yet we know how to manage it from beginning to end just as well as we know how to manage typhoid fever, diphtheria, bronchitis, or any other disease with which we are constantly familiar. Those diseases are managed, and managed successfully, on universally-recognised general principles. Influenza also is managed, and managed successfully, on principles that are similarly recognised. All medical men do not administer one and the same drug, or even order the same identical diet for every individual case of influenza: neither do they all prescribe the same drug and the same diet for every case of bronchitis, or of diphtheria, or of typhoid fever. But though there is diversity in the details of treatment, there is all possible unanimity in the principles. Every influenza patient who submits himself loyally to an intelligent family or consulting physician may feel quite confident that he will be treated on those sound physiological and therapeutic principles that experience has commended to the intelligence of the ablest men in the medical profession.

An anxious public, remembering the facts of the past two winters, and being daily alarmed by the reports of the morning and evening newspapers, is not unnaturally desirous of knowing if anything whatever can be done in the way of prevention. There are two answers to this anxious enquiry. The first is that very little can be done by the mere swallowing of drugs; the second, that much can be done by intelligence and resolution on the part of non-professional persons themselves. The writer, speaking as a physician and as a former victim of influenza, is of opinion that three main factors distinctly tend to encourage an attack of the disease, viz., a gorged state of the liver and other internal parts, an over-worked and depressed condition of the nervous system, and exposure in this gorged and debilitated condition to cold and damp. It is not improbable that a fairly energetic liver pill, with very early rest in bed for a day or two, has averted many an attack of the disease or much diminished its severity. But what of the microbe? asks the reader of a scientific turn of mind. Nobody knows very much about the microbe; but everybody is fully convinced that in certain conditions of the system we are all peculiarly sensitive to epidemic influences, whilst in certain other conditions those influences seem to pass us by as if we were altogether invulnerable. It is not easy to begin mending our habits in mid-winter. But as the epidemic has chosen to visit us in mid-winter, it will be well for us to make some decided efforts at improvement. Those who are still feeling themselves to be in a normal state of health should take reasonable exercise, but avoid fatigue. It would be well for them to take a little alterative medicine under the doctor's direction. They should make their working days as short as possible, and their worries as few and as small as they can. They should avoid exposure to cold and damp, particularly when they are hungry. Those who are already feeling languid and depressed should be doubly careful. They ought to undergo a little alterative treatment under the doctor's direction, and if possible, to stay at home, or even in bed, for a day or two. These simple precautions, we are persuaded, are of great value, and may be practised by all men.

